# Towards in vivo focal cortical dysplasia phenotyping using quantitative MRI

**DOI:** 10.1016/j.nicl.2017.04.017

**Published:** 2017-04-20

**Authors:** Sophie Adler, Sara Lorio, Thomas S. Jacques, Barbora Benova, Roxana Gunny, J. Helen Cross, Torsten Baldeweg, David W. Carmichael

**Affiliations:** a Developmental Neurosciences, UCL Great Ormond Street Institute of Child Health, University College London, London, UK; b Developmental Biology and Cancer Programme, UCL Great Ormond Street Institute of Child Health, University College London, London, UK; c Department of Paediatric Neurology, 2nd Faculty of Medicine, Charles University and Motol University Hospital, Prague, Czech Republic; d 2nd Faculty of Medicine, Charles University, Prague, Czech Republic; e Department of Radiology, Great Ormond Street Hospital for Children, London, UK

**Keywords:** Focal cortical dysplasia, Biophysical tissue properties, Histology, Radiology, MRI, Quantitative mapping, qMRI, Quantitative MRI, Epilepsy surgery, Malformation of cortical development

## Abstract

Focal cortical dysplasias (FCDs) are a range of malformations of cortical development each with specific histopathological features. Conventional radiological assessment of standard structural MRI is useful for the localization of lesions but is unable to accurately predict the histopathological features. Quantitative MRI offers the possibility to probe tissue biophysical properties in vivo and may bridge the gap between radiological assessment and ex-vivo histology. This review will cover histological, genetic and radiological features of FCD following the ILAE classification and will explain how quantitative voxel- and surface-based techniques can characterise these features. We will provide an overview of the quantitative MRI measures available, their link with biophysical properties and finally the potential application of quantitative MRI to the problem of FCD subtyping. Future research linking quantitative MRI to FCD histological properties should improve clinical protocols, allow better characterisation of lesions in vivo and tailored surgical planning to the individual.

## Highlights

• In vivo lesion phenotyping is crucial for FCD prognosis and surgical planning.
• Structural MRI can detect lesions but cannot predict all histological features.
• qMRI provides in vivo biomarkers for biophysical properties linked to histology.
• Multivariate analyses of morphometry and qMRI measures improve FCD phenotyping.

## 1 Introduction

Cortical dysplasias are malformations of brain development that are highly epileptogenic. They are a common cause of drug-resistant focal epilepsy in adults and the most common cause in children, the underlying aetiology in 42% of paediatric epilepsy surgery cases (Harvey et al., 2008). Resective surgery is the most effective treatment to eliminate seizures in drug-resistant focal epilepsy population (Fauser et al., 2004), provided there is a well-characterised epileptic focus.

Focal cortical dysplasia (FCD) encompasses a broad spectrum of histopathological and genetic abnormalities. There have been a number of different classification systems but in 2011 the International League Against Epilepsy (ILAE) developed a three-tiered classification system. According to the ILAE, FCD type I has abnormal radial and/or tangential lamination, FCD type II is also associated with aberrant cytology, specifically dysmorphic neurons, and FCD type III occurs alongside another lesion, e.g. hippocampal sclerosis or tumours (Blumcke et al., 2011). From the genetic perspective FCD subtypes are characterised by a complex interplay between many signalling molecules involved mostly, but not exclusively, in the mTOR pathway.

Non-invasive subtype classification is crucial for several clinical reasons. First, the epileptogenicity of FCD is related to the histopathological features (Boonyapisit et al., 2003). Second, surgical planning is significantly improved when lesions are identified on pre-operative MRI (Téllez-Zenteno et al., 2010) and variability in outcome is seen according to FCD subtypes, with type I and type IIA having poorer post-surgical outcomes than type IIB (Hong et al., 2015, Mühlebner et al., 2011). This may be explained by the fact that those subtypes often present more diffuse or subtle lesions, with poorly defined boundaries (Blumcke et al., 2011, Hong et al., 2015). The ability to characterise FCD subtypes in vivo is therefore clinically relevant as it is informative of post-surgical seizure freedom as well as providing a mechanism for exploring aetiological risk factors and research into cognitive trajectories within specific aetiological subtypes.

Radiological assessments carried out on conventional structural MRI indicate that subtypes may have differentiating MRI features, i.e. they can be distinguished by combinations of morphological and image intensity features (Blumcke et al., 2011). However, visual definition of histological characteristics on pre-surgical MRI is challenging and often inconclusive (Kim et al., 2012).

Two converging areas of imaging research offer the possibility for more specific quantitative measurements of brain structure. The first is computational anatomy, which we define as the automated extraction of morphological features and statistical analysis of grey and white matter maps from MRI images such as T1- and T2-weighted (respectively T1w and T2w), or Fluid Attenuated Inversion Recovery (FLAIR); a T2w image with nulled CSF signal. These morphological features can then be correlated with histopathological and genetic characteristics.

The second area is quantitative MRI, which provides an estimate of the parameters governing the conventional image intensity. Standard structural MRI data, such as T1w and T2w, are able to delineate the brain anatomy but are not specific to tissue property variations. A change in image intensity can be caused by a range of underlying neurobiological processes. Quantitative MRI (qMRI) parameters are specific to tissue structure and biophysical properties at the micrometre scale. These parameters are neuroimaging biomarkers for myelin, water and iron content (Bilgic et al., 2012, Deoni et al., 2015, Schweser et al., 2011), and provide the MRI “fingerprints” (Ma et al., 2013) of brain tissue microstructure (Weiskopf et al., 2013). By linking brain tissue property changes with image intensity, qMRI may assist understanding of the neurobiological mechanisms underlying a change in morphometry.

Until recently, qMRI has not been available in a clinical setting due to scan duration and greater complexity in image reconstruction. However, advances in image acquisition now offer the possibility to probe the in vivo tissue microstructure in a clinical setting (Deoni et al., 2015, Weiskopf et al., 2013) with high signal to noise ratio. Furthermore, the increased availability of high field strength (7 Tesla) MRI will enable MRI to be performed at higher resolution and using new contrast mechanisms (Kozlov and Turner, 2010).

Temporal lobe epilepsy is one of the few conditions where qMRI has been routinely used in a clinical setting to differentiate healthy from sclerotic hippocampi as well as to infer histological and connectivity changes in brain areas. T2 mapping has been shown to significantly outperform FLAIR in differentiating sclerotic from healthy hippocampi (Rodionov et al., 2015). Furthermore, histological analysis of resected brain regions has shown that multiple linear regression models using T1 and T2 mapping can accurately predict neuronal loss in hippocampal subfields (Goubran et al., 2015).

**Fig. 1:**
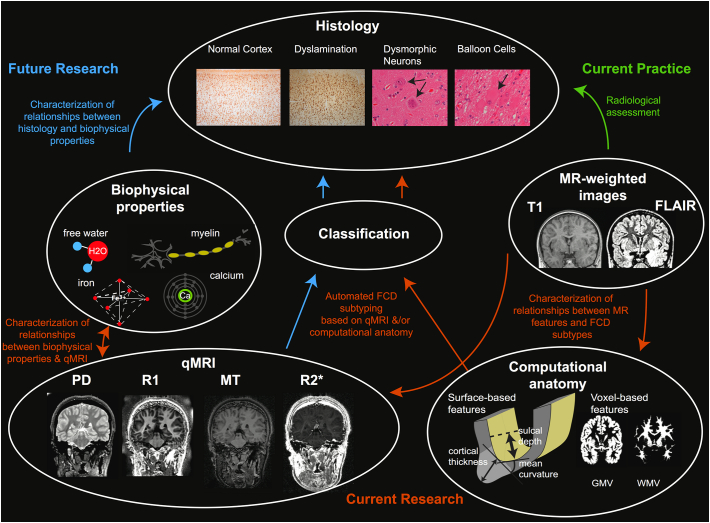
Overview of relationship between histological features, biophysical tissue properties, MR images, computational anatomy measures and quantitative neuroimaging. Green arrow indicates current link between MR-weighted images and ex vivo histology. Orange arrows indicate areas of current research using computational anatomy based on MR-weighted images for FCD subtyping. Blue arrows indicate future directions to characterise biophysical tissue properties of FCDs using qMRI. qMRI maps are sensitive to biophysical tissue properties, such as myelin, iron, calcium and free water content. The qMRI profiles of different FCD subtypes are currently unknown but may offer a technique to probe histology in vivo. (For interpretation of the references to color in this figure legend, the reader is referred to the online version of this chapter.)

The first goal of this review is to report current understanding of histological, genetic and radiological features of FCD lesions. We then investigate the link between those characteristics and the computational anatomy measures reported in the literature. We consider the benefit of using qMRI estimates in the context of FCD subtyping (Fig. 1). Finally, we discuss the need for combinations of computational anatomical features with “in vivo histology” provided by qMRI for non-invasive lesion characterisation.

## 2 Histopathology of focal cortical dysplasia

The first detailed descriptions of FCDs were provided by Taylor and colleagues in 1971 (Taylor et al., 1971). The newest classification scheme, provided by the ILAE, has improved inter-observer agreement among expert neuropathologists (Coras et al., 2012). In this review we focus mainly on FCD types I, IIA and IIB (see Table 1).

**Table 1:** Characteristics of FCD lesion subtypes. The table summarises the histological, genetic and radiological features reported to characterise lesion types.

Lesion subtype	Histology	Genetics	Radiological features
FCD I (A&B)	FCD IA: radial cortical dyslamination (Blumcke et al., 2011) FCD IB: tangential cortical dyslamination (Blumcke et al., 2011)	• Variants in DEPDC5 (Baulac et al., 2015) • FCD IB: chromosomal rearrangement in AKT3 (Conti et al., 2015)	• Cortical thinning • Grey/white matter blurring • Signal abnormalities on T1w/T2w imaging • Lobar hypoplasia (Blumcke et al., 2011, Kabat and Król, 2012, Kim et al., 2012, Krsek et al., 2008)
FCD IIA	Dysmorphic neurons (Blumcke et al., 2011)	• Variants in NPRL3 (Sim et al., 2016) • Variants in DEPDC5 (Mirzaa et al., 2016) • Variants of TSC2 (Majores et al., 2005) • Mutations of mTOR (Lim et al., 2015, Mirzaa et al., 2016) • Mutation in PI3KCA (Jansen et al., 2015)	• Cortical thickening • Grey/white matter blurring • Signal abnormalities on T1w/T2w imaging • Abnormal gyrification (Kabat and Król, 2012, Krsek et al., 2008)
FCD IIB	Dysmorphic neurons and balloon cells (Blumcke et al., 2011)	• Variants of TSC1 (Becker et al., 2002) • Mutations of mTOR (Lim et al., 2015, Mirzaa et al., 2016) • Variant in exon 8 of PTEN (Weiskopf et al., 2013)	• Cortical thickening • Grey/white matter blurring • Signal abnormalities on T1w/T2w imaging • Transmantle sign • Abnormal gyrification (Kim et al., 2012, Mühlebner et al., 2011) (Barkovich et al., 1997)

### 2.1 FCD type I

FCD type I is characterised by isolated lesions in the neocortex with radial (type IA) or tangential (type IB) cortical dyslamination, or a mixture of both (type IC).

In FCD type IA the radial cortical dyslamination manifests with prominent microcolumns (Blumcke et al., 2011). A “microcolumn” is defined as at least 8 neurons aligned in a vertical orientation within the cortex (Rakic, 1988). During cortical development, radial units form that consist of cells that originate from progenitor stem cells, share the same birthplace, migrate along the same pathway and reside in the cortex within the same ontogenetic column. The micro-columnar disorganization in FCD type IA is most prominent within layer 3. These lesions are characterised by a thinner neocortex and higher neuronal densities (Mühlebner et al., 2011). Furthermore, the grey-white matter boundary is usually less easily defined due to increased numbers of heterotopic neurons.

In FCD type IB the normal 6-layered composition of the cortex is disrupted. There may be no identifiable layers in the neocortex, or disruptions may be restricted to abnormal layering of layer 2, layer 4, or both (Blumcke et al., 2011).

### 2.2 FCD type II

In focal cortical dysplasia type II, in addition to disrupted cortical lamination, there are specific cytologic abnormalities. Both FCD types IIA and IIB have dysmorphic neurons characterised by increased size, abnormal orientation and abnormal cytoarchitecture with an indistinct border between the grey matter and white matter.

FCD Type IIB is defined by the presence of balloon cells (Blumcke et al., 2011). These cells have large bodies, eosinophilic cytoplasm, which lacks Nissl substance, and have eccentric, sometimes multiple nuclei (Blumcke et al., 2011). They can be located in any layer of the cortex or in the underlying white matter but are frequently found in the subcortical white matter or in superficial cortical layers. The nature of balloon cells is uncertain but a few studies suggest that they are related to progenitor cells (reviewed by Yasin et al., 2010). Furthermore, they show a defect in the cellular process of autophagy, which is dependent on overactivity of the serine/threonine kinase, mTOR (Yasin et al., 2013). FCD type IIB lesions also exhibit changes in myelination that are hypothesised to result from aberrant oligodendroglial cell differentiation and reduced neuronal cell densities (Mühlebner et al., 2011).

Distinguishing FCD subtypes and differentiating them from other neurodevelopmental abnormalities, on histopathological review can be challenging. Although FCD types IIA and IIB can be consistently differentiated, FCD type I subtypes are more difficult to distinguish and the accurate histopathological classification of these less subtypes is related to the level of expertise and training of the neuropathologist (Coras et al., 2012). Furthermore, heterogeneity in the histopathological appearance of a lesion is often found within an individual further complicating classification.

## 3 Genetics of the focal cortical dysplasias

In recent years, much evidence has accumulated for the common genetic origin of focal epilepsy with FCD (Baulac et al., 2015), with causative pathogenic variants present in the germ line of rare familial cases (see Table 1). An interesting example is the DEPDC5 gene involved both in pathogenesis of a typical genetic epilepsy syndrome (familial focal epilepsy with variable foci – FFEVF) and of FCD (Baulac et al., 2015). DEPDC5 codes for a subunit of GATOR1 complex, an upstream regulator of the mTORC1 protein. In addition, pathogenic variants in the DEPDC5 gene have been identified in a case of hemimegalencephaly with the histological pattern of FCD IIA (Mirzaa et al., 2016). Furthermore, a study of familial cases of histologically confirmed FCD IIA identified germ line pathogenic variants in NPRL3 gene, another mTOR regulator (Sim et al., 2016).

The mTOR pathway has generated great interest in the field of genetic research of various malformations of cortical development, FCD being among them. Indeed, the histological pattern of FCD IIB lesions is identical or closely resembles that of cortical tubers in tuberous sclerosis complex (TSC) and hemimegalencephaly (Majores et al., 2005). Therefore, it has been hypothesised that, given their similarities, these lesions might share a common genetic background. Since pathogenic variants in TSC1 and TSC2 genes have long been known to cause TSC, they have also been studied in FCD lesions, and pathogenic variants of TSC1 gene discovered in FCD IIB brain tissue samples (Becker et al., 2002). Furthermore, interesting allelic variants of TSC2 gene have been observed in FCD IIA samples (Majores et al., 2005), contributing to the evidence for involvement of TSC1 and TSC2 genes, both upstream inhibitors of mTOR signalling. In contrast to these findings, no clear causative pathogenic variants were found for either TSC1 or TSC2 genes in brain tissue samples from a different cohort of FCD patients (Gumbinger et al., 2009). Somatic mutations of mTOR gene itself were discovered in FCD IIA (Lim et al., 2015, Mirzaa et al., 2016) and FCD IIB (Lim et al., 2015) samples.

Located further upstream of the complex PI3K-AKT-MTOR pathway, the AKT3 and PI3KCA genes contribute to mTOR signalling, and these are primarily involved in severe cortical malformations associated with complex megalencephaly and hemimegalencephaly syndromes (Rivière et al., 2012). As previously mentioned, these malformations may share similar histological features with FCD, and we might expect similar genetic background underlying their formation; indeed, a somatic mutation in PI3KCA was detected in an FCD IIA brain tissue sample (Jansen et al., 2015). In addition, somatic missense variant in exon 8 of *PTEN1* was described in an FCD IIB sample (Schick et al., 2006). Furthermore, a chromosomal rearrangement in the region encompassing the AKT3 gene was identified in the brain tissue sample of a dysplastic frontal lobe, showing the histological pattern of FCD IB (Conti et al., 2015).

The aforementioned examples point to the complex interplay between many signalling molecules involved mostly, but not exclusively, in the mTOR pathway. Further study of these molecules and their interactions is warranted if we aim to understand the complex genetic background of formation of FCD.

The intriguing possibility is that the interplay between genetic background and the formation of the cortex may provide defining features of FCD subtypes that can be measured in vivo using qMRI. Previous studies show that genetics affects brain structure both at the cortical and subcortical level (Hibar et al., 2015, Peper et al., 2007). So far, the majority of studies evaluating the correlation of neuroimaging features and genetics in epilepsy have been carried out on mesial temporal lobe epilepsy (MTLE) patients and their asymptomatic first-degree relatives. Increased T2 relaxometry (Suemitsu et al., 2014), morphological alterations (Alhusaini et al., 2016) and microstructural white matter alterations (Whelan et al., 2015) have been discovered in MTLE patients and their asymptomatic siblings, indicative of the heritability of anatomical traits. However, the interrelation between the genetics of FCDs and the neuroimaging phenotype remains to be determined.

## 4 Radiological identifiers of focal cortical dysplasias

On T1w, T2w and FLAIR MRI scans, FCDs of all types often demonstrate abnormal cortical thickness, an indistinct grey-white matter junction, signal abnormalities in the subcortical white matter and grey matter and irregular cortical folding patterns (Bernasconi et al., 2011, Kabat and Król, 2012, Leach et al., 2014a, Leach et al., 2014b, Mellerio et al., 2012). Lesion visibility can change with respect to developing myelination in the brain, the so-called ‘disappearing’ lesion, which may be more obvious on a scan of an unmyelinated brain in infancy but become less distinct later in childhood (Eltze et al., 2005).

### 4.1 FCD type I

MR features that can assist delineation of these often subtle and difficult to discern lesions include cortical thinning, blurring of the grey-white matter boundary and lobar or sublobar hypoplasia which may be accompanied by atrophy of the white matter (Blumcke et al., 2011, Kabat and Król, 2012, Kim et al., 2012, Krsek et al., 2008, Téllez-Zenteno et al., 2010). The cortical thinning may be linked to altered neuronal density in FCD type I (Blumcke et al., 2011). The white matter changes may be visualised by hypointensities on T1w and hyperintensities on T2w imaging. There are no visually identifiable differences between the MR features of types IA and IB. However, the distribution of these two subtypes across the cortex may be different, with type IA more frequently located in the temporal lobe and IB more frequently located in extra-temporal cortex (Kabat and Król, 2012).

### 4.2 FCD type II

Radiological MRI features include grey-white matter blurring, cortical thickening, white matter hypointensities on T1w and increased signal intensity on T2w, FLAIR images as well as abnormal gyrification patterns, often visible as an asymmetric folding pattern (Blumcke et al., 2011).

Radiological features of FCD type IIB are the same as IIA (Kabat and Król, 2012, Krsek et al., 2008). However, FCD type IIB can also demonstrate the distinguishing transmantle sign, a T2w hyperintense signal extending from the subcortical white matter to the lateral ventricle, best seen on FLAIR and proton density images (Barkovich et al., 1997). This white matter signal alteration might reflect the involvement of glial-neuronal bands (Colombo et al., 2009), and is the only MRI feature able to accurately identify FCD type IIB (Kim et al., 2012, Mühlebner et al., 2011).

Increased T2w signal intensity present in the white matter of FCD IIB has been related to the demyelination found histologically, which is associated with severe fibre loss and altered myelin sheaths, abnormal cells and sometimes oedema (Garbelli et al., 2011, Mühlebner et al., 2011, Zucca et al., 2016).

In light of the above, many radiological features are shared by different FCD subtypes (as shown in Table 1), therefore we are currently not able to reliably distinguish subtypes on the basis of conventional visual analysis. The remainder of this review will focus on quantitative MRI features, and their incorporation into automated classification algorithms.

## 5 Computational anatomy

Computerised algorithms can automatically estimate different brain features, such as volume, thickness and shape, relying on the image intensity and tissue contrast to determine anatomical boundaries. Both voxel- and surface-based techniques can be used to extract morphometry and intensity measures from T1w, T2w and FLAIR images to quantify FCD lesion attributes (Guerrini et al., 2015). Voxel-based measures include grey matter and white matter density, also referred to as volume (House et al., 2013, Huppertz et al., 2005), while surface-based features include cortical thickness, sulcal depth, curvature, local gyrification index and intensity sampling (Adler et al., 2016, Ahmed et al., 2015, Hong et al., 2015). These techniques quantify whole-brain structural abnormalities and group-level patterns have been used to dissociate FCD subtypes. For example, surface-based techniques have been used to quantify cortical thinning in FCD type I, and cortical thickening and decreased folding complexity in FCD type II (Hong et al., 2016).

However, overall there is a dearth of computational anatomy findings differentiating lesion subtypes. This may be explained by the fact that current measures derived from T1w and T2w data are affected by a mix of tissue properties that may have confounding effects on tissue contrast and hence reduce the sensitivity of computational anatomy measures. For example, comparisons between histology- and MRI-based measures of cortical thickness generally show good correspondence (Fischl and Dale, 2000, Scholtens et al., 2015, Song et al., 2015). However, MRI-based morphological measures can be confounded by other microstructural changes, such as pronounced cortical myelination in the motor cortex blurring the grey-white boundary and leading to underestimates of cortical thickness (Glasser and Van Essen, 2011, Scholtens et al., 2015). This is particularly problematic in FCDs where blurring is a characteristic abnormality (Blumcke et al., 2011).

## 6 Quantitative MRI (qMRI)

As indicated above, anatomical variations detected from structural MRI data (e.g. T1w, T2w, FLAIR) might be due to true morphological alterations or can be the results of changes in MRI contrast due to biophysical tissue properties, such as water, myelin and iron content, at the microstructural scale (Lorio et al., 2016, Weiskopf et al., 2013). Quantitative MRI provides measures of parameters that can be used as biomarkers of specific tissue properties (Table 2; Tofts, 2005). Here we briefly describe the current range of MRI parameters that can be measured in a clinically relevant timescale (see Table 2). This is limited to MRI features available at mm resolution and therefore excludes MR spectroscopy, which can measure brain metabolites directly typically at a cm scale in the human brain.

**Table 2:** Summary of the main tissue properties affecting different MR parameters.

		**Tissue properties/bio-physical processes**											
		Myelin		Iron		Calcium		Free water		Inflammation (glial cell proliferation)		Neuronal density	
			References		References		References		References		References		References
**MRI parameters**	T1	↓	(Rooney et al., 2007, Stüber et al., 2014, Waehnert et al., 2016)	↓	(Stüber et al., 2014)	↓	(Wehrli, 2013)	↑	(Rooney et al., 2007)				
	PD	↓	(Neeb et al., 2008)					↑	(Tofts, 2005)				
	MT	↑	(Graham and Henkelman, 1999)					↓	(Tofts, 2005)				
	R2*	↑	(Tofts, 2005)	↑	(Haacke et al., 2005, Langkammer et al., 2010, Wehrli, 2013, Yao et al., 2009)			↓	(Haacke et al., 2005)				
	R2	↑	(Briellmann et al., 2002)	↑	(Whittall et al., 1997)					↓	(Briellmann et al., 2002)		
	χ	↓	(Gupta et al., 2001)	↑	(Langkammer et al., 2010, Liu et al., 2011, Yao et al., 2009)	↓	(Schweser et al., 2011)						
	FA	↑	(Beppu et al., 2003, Johansen-Berg and Behrens, 2013)							↓	(Werring et al., 1999)		
	MD	↓	(Alexander et al., 2012)					↑	(Feldman et al., 2010)				
	ICVF	↑	(Jespersen et al., 2010)									↑	(Winston et al., 2014)
	IVF							↑	(Zhang et al., 2012)				
	Micro FA	↑	(Kaden et al., 2016a)							↓	(Werring et al., 1999)		

The symbol ↑ represents the increase of an MR parameter due to the presence of a specific tissue property/biophysical process; the symbol ↓ stands for an MR parameter decrease. T1: longitudinal relaxation time; PD: proton density; MT: magnetisation transfer; R2*: effective transverse relaxation rate; χ: susceptibility mapping; FA: fractional anisotropy; MD: mean diffusivity; ICVF: intra-cellular volume fraction, IVF: isotropic volume fraction.

### 6.1 T1 relaxation time

T1 relaxation time is mainly affected by myelin and water content (Table 2; Rooney et al., 2007, Stüber et al., 2014, Waehnert et al., 2016). For this reason many studies have used T1 relaxometry to map the in vivo myeloarchitecture of the cerebral cortex at 3T and 7T, highlighting densely myelinated primary and extra-striate visual areas (Lutti et al., 2014). Previous studies showed high similarity between T1 value changes across cortical layers and myelin histological staining in those brain regions (Lutti et al., 2014, Waehnert et al., 2016). This underlies the sensitivity of qMRI to subtle variations in tissue properties, particularly at high field strength, and highlights the possibility to perform the parcellation of the cerebral cortex from in vivo MRI data (Lutti et al., 2014, Waehnert et al., 2016). This is particularly relevant for FCD where normal cortical layering is perturbed. In addition, FCD provides an opportunity to evaluate the correspondence between MRI derived cortical structure measurements with histological data in surgical patients.

### 6.2 Magnetization transfer (MT)

Another MRI parameter relevant for FCD lesion characterisation is magnetization transfer (MT) imaging. MT specifically targets the exchange of magnetization between protons bound to macromolecules and water protons and is thought to be the MRI biomarker most specific to myelin content (Table 2; Graham and Henkelman, 1999).

### 6.3 Proton density (PD)

Altered amount of tissue water can be measured using proton density (PD), a parameter sensitive to the density of MRI-visible protons, which are mainly present in tissue water (Table 2; Tofts, 2005). The non-aqueous protons, such as the ones bound to macromolecules, are MRI invisible and do not contribute to the PD signal (Neeb et al., 2008).

### 6.4 Transverse relaxation time (T2)

T2 (= 1 / R2) is the transverse relaxation time, which is related to is related to water mobility, iron deposition, myelin and glial cell count (Table 2; Briellmann et al., 2002). The effective transverse relaxation parameter that describes the signal decay including main magnetic field inhomogeneities is known as T2*. T2 and T2* are related by the following equation:

$$\frac{1}{{T2}^{*}}=\frac{1}{{T2}^{\prime }}+\frac{1}{T2}$$

where T2′ is the additional relaxation related to the presence of inhomogeneities.

T2* is sensitive to local magnetic field perturbations caused by the presence of paramagnetic substances such as iron. Previous studies demonstrated high correlation of the effective transverse relaxation rate R2* (= 1 / T2*) with iron concentration measured in ferritin-rich structures (Table 2; Langkammer et al., 2010, Yao et al., 2009). However R2* is also sensitive to global sources of field perturbations, such as those from air-water interfaces. Those perturbations cause strong signal changes unrelated to microstructure in basal temporal and frontal brain regions.

### 6.5 Phase-based susceptibility MRI

Phase-based susceptibility MRI provides images sensitive to the local microscopic magnetic agents while removing global sources of magnetic field perturbations increasing their potential specificity to microstructural tissue properties (Table 2). These techniques can distinguish between paramagnetic haemorrhage and diamagnetic calcification (Gupta et al., 2001). Molecular magnetic dipoles (e.g. ferritin core) aligning to the static magnetic field, increase the local susceptibility depending on dipole strength and concentration, while highly myelinated fibres exhibits the lowest susceptibilities in brain (Langkammer et al., 2010, Liu et al., 2011, Yao et al., 2009).

### 6.6 Diffusion MRI

The MRI signal can be sensitised to the microscopic movement of water self-diffusion, that is the random translational motion of water molecules (Johansen-Berg and Behrens, 2013). In tissue, diffusion is hindered by the semipermeable cell membranes, which couple the diffusivity in extra- and intracellular sub-spaces. Over the last 20 years diffusion has been based on a tensor model of unhindered diffusion allowing for the estimation of diffusion parameters such as fractional anisotropy (FA) and mean diffusivity (MD). FA characterises the directional preference (most preferred/least preferred) of water diffusion in tissue. The presence of highly organised fibres and cell proliferation increase FA, as the water molecules move along the fibre direction (Table 2; Beppu et al., 2003, Johansen-Berg and Behrens, 2013). Whereas axonal directional dispersion, cellular inflammation and demyelination decrease FA, as water molecules tend to move in all directions (Table 2; Basser and Pierpaoli, 2011, Johansen-Berg and Behrens, 2013, Werring et al., 1999).

MD reflects the average magnitude of molecular displacement by diffusion. It can be seen as an inverse measure of the membrane density. Brain oedema and demyelination increase MD values, whereas cell proliferation and white matter maturation reduce MD (Table 2; Alexander et al., 2012, Feldman et al., 2010).

Both FA and MD values are affected by fibre crossings and orientation dispersion, ubiquitous brain features, which confound the relation with other patho-physiological phenomena. This hampers the neurobiological interpretation of signal abnormalities, as they can be due to microsctructural changes or can be caused by neural circuitry variations resulting in a modified distribution of the neurite orientation.

A robust biomarker for cortical and subcortical microstructure needs to be unaffected by orientation dispersion and crossing fibres (Kaden et al., 2016a), which naturally differ between subjects. However, both FA and MD values are affected by these confounding factors, hampering the neurobiological interpretation of signal abnormalities. Another limitation of standard FA and MD maps is that they do not account for the presence of different tissue components, such as neurons, microglia and extracellular space, across which diffusion parameters differ (Kaden et al., 2016a).

Recent improvements in data acquisition and modelling, such as neurite orientation dispersion and density imaging (NODDI) (Zhang et al., 2012) and multi-compartment microscopic diffusion (Kaden et al., 2016a), enable the separation of effects of fibre crossings and orientation dispersion within different tissue compartments (Kaden et al., 2016a, Kaden et al., 2016b, Zhang et al., 2012), allowing more specific estimation of diffusion parameters.

The intra-cellular volume fraction of NODDI represents the space within the membrane of neurites (Table 2). The signal relative to this environment is further characterised by neurite orientation, a feature distinguishing highly coherently oriented white matter structures, white matter areas with bending and fanning axons, cortical and subcortical grey matter structures composed by sprawling dendritic processes in all directions (Zhang et al., 2012, Sotiropoulos et al., 2012). The extra-cellular volume fraction represents the space around neurites, where glial cells and cell bodies, in grey matter, are present (Table 2; Zhang et al., 2012). In this space, the signal is characterised with anisotropic Gaussian diffusion model, as the presence of neurites hinders water molecules diffusion (Zhang et al., 2012). The CSF compartment, referring to the space occupied by cerebrospinal fluid, allows to minimize the confounding effect of CSF-contamination, especially in the periventricular white matter regions (Zhang et al., 2012).

The microscopic FA of the multi-compartment model provides separate measures for the diffusion anisotropy of water molecules inside and outside the neurites, as the two pools have different contribution to the diffusion signal (Table 2; Kaden et al., 2016a). Unlike the standard FA metrics, this novel parameter is invariant to the fibres' orientation, which physiologically varies between subjects, thus microscopic FA can improve the detection of microstructural abnormalities. Microscopic FA has been recently proved to be a sensitive biomarker for microstructural changes associated to TSC in an animal model (Kaden et al., 2016a).

The majority of diffusion models commonly used to disentangle the effects of fibre crossings and orientation dispersion, rely on strong assumptions or constraints. When the tissue properties significantly differ from those constraints, the model can lead to erroneous interpretations, as might be the case in cortical grey matter (Lampinen et al., 2017). Despite the criticisms raised regarding the model assumptions (Lampinen et al., 2017), NODDI is frequently used to study the human brain cortex (Colgan et al., 2016, Eaton-Rosen et al., 2015, Nazeri et al., 2016). In the context of FCD characterisation, it may be necessary to refine diffusion models to encompass the range of brain tissue microstructure present in the cortex. However, it must be noted that there is a limit to the model complexity that current diffusion data can support. For clinical purposes a robust model, even if biophysically inaccurate for grey matter, may yield consistent signal changes related to FCD pathology which in turn could aid diagnostic specificity.

#### 6.6.1 Conclusion on qMRI

qMRI parameters are more specific than standard MRI to biophysical tissue properties changes. However multiple microstructural properties can elicit changes in each parameter's values, limiting its specificity. The combination of multiple quantitative parameters offers an approach to investigate and disentangle the contribution of different tissue properties to the MRI signal, allowing a more detailed characterisation of the whole brain using non-invasive techniques (“in vivo histology”) (Dinse et al., 2015, Stüber et al., 2014). For this goal to be realised, determining the multivariate relationship between tissue properties and qMRI parameters is an essential step (Mohammadi et al., 2015). FCD offers a unique opportunity for validation by performing these MRI measurements in the human brain with the subsequent analysis of surgically resected tissue (Fig. 1).

## 7 Quantitative MRI in focal cortical dysplasia detection

In FCD, qMRI has been used in lesion detection as well as region of interest (ROI) studies to histologically characterise and subtype lesions. In terms of lesion detection, diffusion tensor imaging shows decreased subcortical fibre connectivity in and around the region affected by FCD (Lee et al., 2004) and reduced intra-cellular volume fraction (ICVF) in the lesion area (Winston et al., 2014). These findings are in agreement with altered diffusion measures performed on histology samples (Vargova et al., 2011). FA and MD are altered in the subcortical white matter subjacent to the FCD as well as beyond the MR-visible abnormality (Widjaja et al., 2009). However, alterations in FA and MD distant from the FCD have also been reported (Eriksson et al., 2001, de la Roque et al., 2005) and thus the general consensus is these features are not specific enough for lesion classification. Furthermore, changes in diffusion measures/markers have been discovered in other malformations of cortical development as well as in epilepsy patients with normal MRI (Eriksson et al., 2001, Rugg-Gunn et al., 2001). However, future work will be required to evaluate whether more specific diffusion measures such as ICVF provide a greater utility for FCD lesion classification.

ROI studies have estimated the correlation between histology measures and MRI parameters (Reeves et al., 2015). MT, T1, T2 and T2* values are correlated with myelin alteration in the lesion/perilesional areas. FCD type IIA shows decreased T2 and T2* values with respect to the mean values for normal cortex (Reeves et al., 2015). Overall however, the qMRI signature of FCD subtypes is not yet well defined, mainly due to the lack of quantitative histopathology information as well as incomplete knowledge about the relationship between genetics, histopathology and qMRI parameters (Mühlebner et al., 2011). The existing literature presents data in small patient samples, often limited to a specific diagnosis (i.e. specific subtypes), which increases the chance of bias and hampers generalisation.

## 8 Combinations of qMRI and morphometry measures in FCD

Individual qMRI parameters are unlikely to distinguish subtypes or even detect all FCD lesions, as they are characterised by subtle and overlapping features. As brain structure may be profoundly or subtly affected by the interplay of genetic and histological factors, successful approaches are likely to entail the design of integrated methods to model morphology, intensity and quantitative signals in a multivariate framework.

**Fig. 2:**
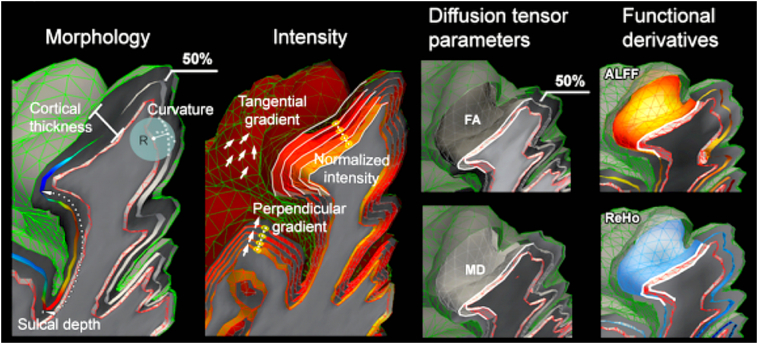
Computational and quantitative MRI features used by Hong et al., Morphology features include cortical thickness and sulcal depth. Intensity features include normalized T1 and FLAIR signal intensity sampled at multiple intra- and sub-cortical surfaces, as well as horizontal and vertical intensity gradients. Diffusion tensor based features include fractional anisotropy (FA) and mean diffusivity (MD). Resting-state fMRI features include amplitude of local functional fluctuation (ALFF) and regional homogeneity (ReHo).

Adapted from “MRI-Based Lesion Profiling of Epileptogenic Cortical Malformations” by Hong et al., 2015. Adapted with permission.

Hong and colleagues combined quantitative MRI, morphological and functional MRI features in a multivariate analysis using machine learning algorithms (Hong et al., 2015, Hong et al., 2017) to classify lesion subtypes (Fig. 2). The study used a surface-based analysis framework to include anatomical, diffusion, and resting-state functional MRI (rsfMRI) measures, benefitting from their covariance for improved lesion profiling. Morphology features included cortical thickness and sulcal depth. Intensity features included normalized T1-weighted and FLAIR signal intensity as well as tangential and perpendicular intensity gradients. Diffusion measures included fractional anisotropy and mean diffusivity. Resting state fMRI features included amplitude of low frequency fluctuations (ALFF) in the 0.01–0.08 Hz band, and regional homogeneity (ReHo), a measure of the similarity between one voxel's time series and its neighbours. Anomalies in FCD Type IIB were distributed throughout the cortex and in the subcortical white matter, whereas those in Type IIA clustered at the cortico-subcortical interface (Hong et al., 2015). A supervised classifier was reported to predict the FCD subtype with 91% accuracy, exemplifying the ability of combinations of qMRI and computational anatomical metrics to accurately subtype FCD lesions.

FCDs are often associated with diffuse changes (Hong et al., 2016) in the cortex and also in the white matter. One crucial advantage of in vivo qMRI and morphometry is that they can provide whole-brain measures that can be used to identify lesional and extra-lesional characteristics of FCD subtypes within cortex and white matter. One challenge for future studies will be in distinguishing true malformations of cortical development from other secondary changes such as gliosis, and is likely to require the correlation of computational or qMRI features with the underlying histology. This will be integral in accurately delineating the margins of the lesion for pre-surgical evaluation, intra-operative guidance and patient counselling.

## 9 Conclusions and future directions

The radiological literature regarding FCD subtyping highlights the fact that it is impossible to reliably subtype a lesion based on one MRI feature. However, radiologists are often able to estimate the histopathological subtype with greater than chance accuracy. This suggests that the combination of multiple features may improve accuracy of in vivo classification. Technological advances have shown that it is increasingly possible to map quantitative MRI parameters in clinically feasible scan times. At the same time the correlation between qMRI parameters and tissue microstructure is the subject of intensive investigation. This combined with computational approaches that allow for the comparison of a wide range of imaging features at a local and global level bring the possibility of MRI tissue classification closer to the clinical domain.

FCD is a unique pathology that offers a continuum of tissue organization and distinct changes to neuronal and glial cell types, as well as access to histological classification close to the time point of imaging. Moreover the highly complex structure of the brain is strongly shaped by genetic influences, with several genetic variants underlying differences in lesion subtypes at the individual level (Conti et al., 2015, Jansen et al., 2015, Mirzaa et al., 2016, Sim et al., 2016). On the one hand multivariate analysis of qMRI and computational anatomy seems well placed to characterise FCD lesion type non-invasively while on the other histopathological and genetic analyses of the surgical resections offer the opportunity to validate the sensitivity and specificity of qMRI to tissue properties. The first studies using multivariate analysis for lesion subtyping are now being published. Future studies will need to include multiple quantitative and morphological features in large datasets representative of the entire ILAE classification system. The creation of open source databases of different image modalities and FCD subtypes would facilitate the ability to conduct large-scale studies, create new MR features and test machine learning capabilities. This may open an avenue for new MRI methods to be incorporated into clinical decision making; allowing non-invasive characterisation of the lesion and more individualized surgical planning.

## Disclosure

None of the authors have any conflict of interest to disclose. We confirm that we have read the journal's position on issues involved in ethical publication and affirm that this report is consistent with those guidelines.

## Funding and acknowledgments

This research was supported by the National Institute for Health Research Biomedical Research Centre at Great Ormond Street Hospital for Children NHS Foundation Trust, the Henry Smith Charity and Action Medical Research (GN2214). SA received funding from the Rosetrees Trust. TSJ receives support from The Brain Tumour Charity, Great Ormond Street Children's Charity and Children with Cancer. We would like to thank Seok-Jun Hong for providing useful feedback on the manuscript.

## References

[bb0005] Adler S., Wagstyl K., Gunny R., Ronan L., Carmichael D., Cross J.H., Fletcher P.C., Baldeweg T. (2016). Novel surface features for automated detection of focal cortical dysplasias in paediatric epilepsy. Neuroimage. Clin..

[bb0010] Ahmed B., Brodley C.E., Blackmon K.E., Kuzniecky R., Barash G., Carlson C., Quinn B.T., Doyle W., French J., Devinsky O., Thesen T. (2015). Cortical feature analysis and machine learning improves detection of “MRI-negative” focal cortical dysplasia. Epilepsy Behav..

[bb0015] Alexander A.L., Hurley S.A., Samsonov A.A., Adluru N., Hosseinbor A.P., Mossahebi P., Tromp do P.M., Zakszewski E., Field A.S. (2012). Characterization of cerebral white matter properties using quantitative magnetic resonance imaging stains. Brain Connect..

[bb0020] Alhusaini S., Whelan C.D., Doherty C.P., Delanty N., Fitzsimons M., Cavalleri G.L. (2016). Temporal cortex morphology in mesial temporal lobe epilepsy patients and their asymptomatic siblings. Cereb. Cortex.

[bb0025] Barkovich A.J., Kuzniecky R.I., Bollen A.W., Grant P.E. (1997). Focal transmantle dysplasia: a specific malformation of cortical development. Neurology.

[bb9815] Basser P.J., Pierpaoli C. (2011). Microstructural and physiological features of tissues elucidated by quantitative-diffusion-tensor MRI. J. Magn. Reson..

[bb0030] Baulac S., Ishida S., Marsan E., Miquel C., Biraben A., Nguyen D.K., Nordli D., Cossette P., Nguyen S., Lambrecq V., Vlaicu M., Daniau M., Bielle F., Andermann E., Andermann F., Leguern E., Chassoux F., Picard F. (2015). Familial focal epilepsy with focal cortical dysplasia due to DEPDC5 mutations. Ann. Neurol..

[bb0035] Becker A.J., Urbach H., Scheffler B., Baden T., Normann S., Lahl R., Pannek H.W., Tuxhorn I., Elger C.E., Schramm J., Wiestler O.D., Blumcke I. (2002). Focal cortical dysplasia of Taylor's balloon cell type: mutational analysis of the TSC1 gene indicates a pathogenic relationship to tuberous sclerosis. Ann. Neurol..

[bb0040] Beppu T., Inoue T., Shibata Y., Kurose A., Arai H., Ogasawara K., Ogawa A., Nakamura S., Kabasawa H. (2003). Measurement of fractional anisotropy using diffusion tensor MRI in supratentorial astrocytic tumors. J. Neuro-Oncol..

[bb0045] Bernasconi A., Bernasconi N., Bernhardt B.C., Schrader D. (2011). Advances in MRI for “cryptogenic” epilepsies. Nat. Rev. Neurol..

[bb0050] Bilgic B., Pfefferbaum A., Rohlfing T., Sullivan E.V., Adalsteinsson E. (2012). MRI estimates of brain iron concentration in normal aging using quantitative susceptibility mapping. NeuroImage.

[bb0055] Blumcke I., Thom M., Aronica E., Armstrong D.D., Vinters H.V., Palmini A., Jacques T.S., Avanzini G., Barkovich A.J., Battaglia G., Becker A., Cepeda C., Cendes F., Colombo N., Crino P., Cross J.H., Delalande O., Dubeau F., Duncan J., Guerrini R., Kahane P., Mathern G., Najm I., Ozkara C., Raybaud C., Represa A., Roper S.N., Salamon N., Schulze-Bonhage A., Tassi L., Vezzani A., Spreafico R. (2011). The clinicopathologic spectrum of focal cortical dysplasias: a consensus classification proposed by an ad hoc Task Force of the ILAE Diagnostic Methods Commission. Epilepsia.

[bb0060] Boonyapisit K., Najm I., Klem G., Ying Z., Burrier C., LaPresto E., Nair D., Bingaman W., Prayson R., Lüders H. (2003). Epileptogenicity of focal malformations due to abnormal cortical development: direct electrocorticographic-histopathologic correlations. Epilepsia.

[bb0065] Briellmann R.S., Kalnins R.M., Berkovic S.F., Jackson G.D. (2002). Hippocampal pathology in refractory temporal lobe epilepsy T2-weighted signal change reflects dentate gliosis. Neurology.

[bb0070] Colgan N., Siow B., O'Callaghan J.M., Harrison I.F., Wells J.A., Holmes H.E., Ismail O., Richardson S., Alexander D.C., Collins E.C., Fisher E.M., Johnson R., Schwarz A.J., Ahmed Z., O'Neill M.J., Murray T.K., Zhang H., Lythgoe M.F. (2016). Application of neurite orientation dispersion and density imaging (NODDI) to a tau pathology model of Alzheimer's disease. NeuroImage.

[bb0075] Colombo N., Salamon N., Raybaud C., Ozkara C., Barkovich A.J. (2009). Imaging of malformations of cortical development. Epileptic Disord..

[bb0080] Conti V., Pantaleo M., Barba C., Baroni G., Mei D., Buccoliero A.M., Giglio S., Giordano F., Baek S.T., Gleeson J.G., Guerrini R. (2015). Focal dysplasia of the cerebral cortex and infantile spasms associated with somatic 1q21.1-q44 duplication including the AKT3 gene. Clin. Genet..

[bb0085] Coras R., de Boer O.J., Armstrong D., Becker A., Jacques T.S., Miyata H., Thom M., Vinters H.V., Spreafico R., Oz B., Marucci G., Pimentel J., Mühlebner A., Zamecnik J., Buccoliero A.M., Rogerio F., Streichenberger N., Arai N., Bugiani M., Vogelgesang S., Macaulay R., Salon C., Hans V., Polivka M., Giangaspero F., Fauziah D., Kim J.-H., Liu L., Dandan W., Gao J., Lindeboom B., Blumcke I., Aronica E. (2012). Good interobserver and intraobserver agreement in the evaluation of the new ILAE classification of focal cortical dysplasias. Epilepsia.

[bb0090] de la Roque A.D., Oppenheim C., Chassoux F., Rodrigo S., Beuvon F., Daumas-Duport C., Devaux B., Meder J.-F. (2005). Diffusion tensor imaging of partial intractable epilepsy. Eur. Radiol..

[bb0095] Deoni S.C.L., Dean D.C., Remer J., Dirks H., O'Muircheartaigh J. (2015). Cortical maturation and myelination in healthy toddlers and young children. NeuroImage.

[bb0100] Dinse J., Härtwich N., Waehnert M.D., Tardif C.L., Schäfer A., Geyer S., Preim B., Turner R., Bazin P.L. (2015). A cytoarchitecture-driven myelin model reveals area-specific signatures in human primary and secondary areas using ultra-high resolution in-vivo brain MRI. NeuroImage.

[bb0105] Eaton-Rosen Z., Melbourne A., Orasanu E., Cardoso M.J., Modat M., Bainbridge A., Kendall G.S., Robertson N.J., Marlow N., Ourselin S. (2015). Longitudinal measurement of the developing grey matter in preterm subjects using multi-modal MRI. NeuroImage.

[bb0110] Eltze C.M., Chong W.K., Bhate S., Harding B., Neville B.G.R., Cross J.H. (2005). Taylor-type focal cortical dysplasia in infants: some MRI lesions almost disappear with maturation of myelination. Epilepsia.

[bb0115] Eriksson S.H., Rugg-Gunn F.J., Symms M.R., Barker G.J., Duncan J.S. (2001). Diffusion tensor imaging in patients with epilepsy and malformations of cortical development. Brain.

[bb0120] Fauser S., Schulze-Bonhage A., Honegger J., Carmona H., Huppertz H.-J., Pantazis G., Rona S., Bast T., Strobl K., Steinhoff B.J., Korinthenberg R., Rating D., Volk B., Zentner J. (2004). Focal cortical dysplasias: surgical outcome in 67 patients in relation to histological subtypes and dual pathology. Brain.

[bb0125] Feldman H.M., Yeatman J.D., Lee E.S., Barde L.H.F., Gaman-Bean S. (2010). Diffusion tensor imaging: a review for pediatric researchers and clinicians. J. Dev. Behav. Pediatr..

[bb0130] Fischl B., Dale A.M. (2000). Measuring the thickness of the human cerebral cortex from magnetic resonance images. Proc. Natl. Acad. Sci..

[bb0135] Garbelli R., Zucca I., Milesi G., Mastropietro A., D'Incerti L., Tassi L., Colombo N., Marras C., Villani F., Minati L., Spreafico R. (2011). Combined 7-T MRI and histopathologic study of normal and dysplastic samples from patients with TLE. Neurology.

[bb0140] Glasser M.F., Van Essen D.C. (2011). Mapping human cortical areas in vivo based on myelin content as revealed by T1- and T2-weighted MRI. J. Neurosci..

[bb0145] Goubran M., Bernhardt B.C., Cantor-Rivera D., Lau J.C., Blinston C., Hammond R.R., de Ribaupierre S., Burneo J.G., Mirsattari S.M., Steven D.A., Parrent A.G., Bernasconi A., Bernasconi N., Peters T.M., Khan A.R. (2015). In vivo MRI signatures of hippocampal subfield pathology in intractable epilepsy. Hum. Brain Mapp..

[bb0150] Graham S.J., Henkelman R.M. (1999). Pulsed magnetization transfer imaging: evaluation of Technique1. Radiology.

[bb0155] Guerrini R., Duchowny M., Jayakar P., Krsek P., Kahane P., Tassi L., Melani F., Polster T., Andre V.M., Cepeda C., Krueger D.A., Cross J.H., Spreafico R., Cosottini M., Gotman J., Chassoux F., Ryvlin P., Bartolomei F., Bernasconi A., Stefan H., Miller I., Devaux B., Najm I., Giordano F., Vonck K., Barba C., Blumcke I. (2015). Diagnostic methods and treatment options for focal cortical dysplasia. Epilepsia.

[bb0160] Gumbinger C., Rohsbach C.B., Schulze-Bonhage A., Korinthenberg R., Zentner J., Häffner M., Fauser S. (2009). Focal cortical dysplasia: a genotype-phenotype analysis of polymorphisms and mutations in the TSC genes. Epilepsia.

[bb0165] Gupta R.K., Rao S.B., Jain R., Pal L., Kumar R., Venkatesh S.K., Rathore R.K.S. (2001). Differentiation of calcification from chronic hemorrhage with corrected gradient Echo phase imaging. J. Comput. Assist. Tomogr..

[bb0170] Haacke E.M., Cheng N.Y.C., House M.J., Liu Q., Neelavalli J., Ogg R.J., Khan A., Ayaz M., Kirsch W., Obenaus A. (2005). Imaging iron stores in the brain using magnetic resonance imaging. Magn. Reson. Imaging.

[bb0175] Harvey A.S., Cross J.H., Shinnar S., Mathern G.W., Mathern B.W., ILAE Pediatric Epilepsy Surgery Survey Taskforce (2008). Defining the spectrum of international practice in pediatric epilepsy surgery patients. Epilepsia.

[bb0180] Hibar D.P., Stein J.L., Renteria M.E., Arias-Vasquez A., Desrivières S., Jahanshad N., Toro R., Wittfeld K., Abramovic L., Andersson M., Aribisala B.S., Armstrong N.J., Bernard M., Bohlken M.M., Boks M.P., Bralten J., Brown A.A., Chakravarty M.M., Chen Q., Ching C.R.K., Cuellar-Partida G., den Braber A., Giddaluru S., Goldman A.L., Grimm O., Guadalupe T., Hass J., Woldehawariat G., Holmes A.J., Hoogman M., Janowitz D., Jia T., Kim S., Klein M., Kraemer B., Lee P.H., Olde Loohuis L.M., Luciano M., Macare C., Mather K.A., Mattheisen M., Milaneschi Y., Nho K., Papmeyer M., Ramasamy A., Risacher S.L., Roiz-Santiañez R., Rose E.J., Salami A., Sämann P.G., Schmaal L., Schork A.J., Shin J., Strike L.T., Teumer A., van Donkelaar M.M.J., van Eijk K.R., Walters R.K., Westlye L.T., Whelan C.D., Winkler A.M., Zwiers M.P., Alhusaini S., Athanasiu L., Ehrlich S., Hakobjan M.M.H., Hartberg C.B., Haukvik U.K., Heister A.J.G.A.M., Hoehn D., Kasperaviciute D., Liewald D.C.M., Lopez L.M., Makkinje R.R.R., Matarin M., Naber M.A.M., McKay D.R., Needham M., Nugent A.C., Pütz B., Royle N.A., Shen L., Sprooten E., Trabzuni D., van der Marel S.S.L., van Hulzen K.J.E., Walton E., Wolf C., Almasy L., Ames D., Arepalli S., Assareh A.A., Bastin M.E., Brodaty H., Bulayeva K.B., Carless M.A., Cichon S., Corvin A., Curran J.E., Czisch M., de Zubicaray G.I., Dillman A., Duggirala R., Dyer T.D., Erk S., Fedko I.O., Ferrucci L., Foroud T.M., Fox P.T., Fukunaga M., Gibbs J.R., Göring H.H.H., Green R.C., Guelfi S., Hansell N.K., Hartman C.A., Hegenscheid K., Heinz A., Hernandez D.G., Heslenfeld D.J., Hoekstra P.J., Holsboer F., Homuth G., Hottenga J.-J., Ikeda M., Jack C.R., Jenkinson M., Johnson R., Kanai R., Keil M., Kent J.W., Kochunov P., Kwok J.B., Lawrie S.M., Liu X., Longo D.L., McMahon K.L., Meisenzahl E., Melle I., Mohnke S., Montgomery G.W., Mostert J.C., Mühleisen T.W., Nalls M.A., Nichols T.E., Nilsson L.G., Nöthen M.M., Ohi K., Olvera R.L., Perez-Iglesias R., Pike G.B., Potkin S.G., Reinvang I., Reppermund S., Rietschel M., Romanczuk-Seiferth N., Rosen G.D., Rujescu D., Schnell K., Schofield P.R., Smith C., Steen V.M., Sussmann J.E., Thalamuthu A., Toga A.W., Traynor B.J., Troncoso J., Turner J.A., Valdés Hernández M.C., van 't Ent D., van der Brug M., van der Wee N.J.A., van Tol M.-J., Veltman D.J., Wassink T.H., Westman E., Zielke R.H., Zonderman A.B., Ashbrook D.G., Hager R., Lu L., McMahon F.J., Morris D.W., Williams R.W., Brunner H.G., Buckner R.L., Buitelaar J.K., Cahn W., Calhoun V.D., Cavalleri G.L., Crespo-Facorro B., Dale A.M., Davies G.E., Delanty N., Depondt C., Djurovic S., Drevets W.C., Espeseth T., Gollub R.L., Ho B.-C., Hoffmann W., Hosten N., Kahn R.S., le Hellard S., Meyer-Lindenberg A., Müller-Myhsok B., Nauck M., Nyberg L., Pandolfo M., Penninx B.W.J.H., Roffman J.L., Sisodiya S.M., Smoller J.W., van Bokhoven H., van Haren N.E.M., Völzke H., Walter H., Weiner M.W., Wen W., White T., Agartz I., Andreassen O.A., Blangero J., Boomsma D.I., Brouwer R.M., Cannon D.M., Cookson M.R., de Geus E.J.C., Deary I.J., Donohoe G., Fernández G., Fisher S.E., Francks C., Glahn D.C., Grabe H.J., Gruber O., Hardy J., Hashimoto R., Hulshoff Pol H.E., Jönsson E.G., Kloszewska I., Lovestone S., Mattay V.S., Mecocci P., McDonald C., McIntosh A.M., Ophoff R.A., Paus T., Pausova Z., Ryten M., Sachdev P.S., Saykin A.J., Simmons A., Singleton A., Soininen H., Wardlaw J.M., Weale M.E., Weinberger D.R., Adams H.H.H., Launer L.J., Seiler S., Schmidt R., Chauhan G., Satizabal C.L., Becker J.T., Yanek L., van der Lee S.J., Ebling M., Fischl B., Longstreth W.T., Greve D., Schmidt H., Nyquist P., Vinke L.N., van Duijn C.M., Xue L., Mazoyer B., Bis J.C., Gudnason V., Seshadri S., Ikram M.A., Alzheimer's Disease Neuroimaging Initiative, CHARGE Consortium, EPIGEN, IMAGEN, SYS, Martin N.G., Wright M.J., Schumann G., Franke B., Thompson P.M., Medland S.E. (2015). Common genetic variants influence human subcortical brain structures. Nature.

[bb0185] Hong S.J., Bernhardt B.C., Schrader D., Caldairou B., Bernasconi A., Bernasconi N. (2015). MRI-based lesion profiling of epileptogenic cortical malformations. MICCAI.

[bb0190] Hong S.-J., Bernhardt B.C., Schrader D.S., Bernasconi N., Bernasconi A. (2016). Whole-brain MRI phenotyping in dysplasia-related frontal lobe epilepsy. Neurology.

[bb0195] Hong S.-J., Bernhardt B.C., Caldairou B., Hall J.A., Guiot M.C., Schrader D., Bernasconi N., Bernasconi A. (2017). Multimodal MRI profiling of focal cortical dysplasia type II. Neurology.

[bb0200] House P.M., Lanz M., Holst B., Martens T., Stodieck S., Huppertz H.-J. (2013). Comparison of morphometric analysis based on T1- and T2-weighted MRI data for visualization of focal cortical dysplasia. Epilepsy Res..

[bb0205] Huppertz H.-J., Grimm C., Fauser S., Kassubek J., Mader I., Hochmuth A., Spreer J., Schulze-Bonhage A. (2005). Enhanced visualization of blurred gray–white matter junctions in focal cortical dysplasia by voxel-based 3D MRI analysis. Epilepsy Res..

[bb0210] Jansen L.A., Mirzaa G.M., Ishak G.E., O'Roak B.J., Hiatt J.B., Roden W.H., Gunter S.A., Christian S.L., Collins S., Adams C., Rivière J.-B., St-Onge J., Ojemann J.G., Shendure J., Hevner R.F., Dobyns W.B. (2015). PI3K/AKT pathway mutations cause a spectrum of brain malformations from megalencephaly to focal cortical dysplasia. Brain.

[bb0215] Jespersen S.N., Bjarkam C.R., Nyengaard J.R., Chakravarty M.M., Hansen B., Vosegaard T., Østergaard L., Yablonskiy D., Nielsen N.C., Vestergaard-Poulsen P. (2010). Neurite density from magnetic resonance diffusion measurements at ultrahigh field: comparison with light microscopy and electron microscopy. NeuroImage.

[bb0220] Johansen-Berg H., Behrens T. (2013). Diffusion MRI: From Quantitative Measurement to in Vivo Neuroanatomy.

[bb0225] Kabat J., Król P. (2012). Focal cortical dysplasia – review. Pol. J. Radiol..

[bb0230] Kaden E., Kelm N.D., Carson R.P., Does M.D., Alexander D.C. (2016). Multi-compartment microscopic diffusion imaging. NeuroImage.

[bb0235] Kaden E., Kruggel F., Alexander D.C. (2016). Quantitative mapping of the per-axon diffusion coefficients in brain white matter. Magn. Reson. Med..

[bb0240] Kim D.W., Kim S., Park S.-H., Chung C.-K., Lee S.K. (2012). Comparison of MRI features and surgical outcome among the subtypes of focal cortical dysplasia. Seizure.

[bb0245] Kozlov M., Turner R. (2010). A Comparison of Ansoft HFSS and CST Microwave Studio Simulation Software for Multi-Channel Coil Design and SAR Estimation at 7T MRI.

[bb0250] Krsek P., Maton B., Korman B., Pacheco-Jacome E., Jayakar P., Dunoyer C., Rey G., Morrison G., Ragheb J., Vinters H.V., Resnick T., Duchowny M. (2008). Different features of histopathological subtypes of pediatric focal cortical dysplasia. Ann. Neurol..

[bb0255] Lampinen B., Szczepankiewicz F., Mårtensson J., van Westen D., Sundgren P.C., Nilsson M. (2017). Neurite density imaging versus imaging of microscopic anisotropy in diffusion MRI: a model comparison using spherical tensor encoding. NeuroImage.

[bb0260] Langkammer C., Krebs N., Goessler W., Scheurer E., Ebner F., Yen K., Fazekas F., Ropele S. (2010). Quantitative MR imaging of brain iron: a postmortem validation Study1. Radiology.

[bb0265] Leach J.L., Greiner H.M., Miles L., Mangano F.T. (2014). Imaging spectrum of cortical dysplasia in children. Semin. Roentgenol..

[bb0270] Leach J.L., Miles L., Henkel D.M., Greiner H.M., Kukreja M.K., Holland K.D., Rose D.F., Zhang B., Mangano F.T. (2014). Magnetic resonance imaging abnormalities in the resection region correlate with histopathological type, gliosis extent, and postoperative outcome in pediatric cortical dysplasia. J. Neurosurg. Pediatr..

[bb0275] Lee S.-K., Kim D.I., Mori S., Kim J., Kim H.D., Heo K., Lee B.I. (2004). Diffusion tensor MRI visualizes decreased subcortical fiber connectivity in focal cortical dysplasia. NeuroImage.

[bb0280] Lim J.S., Kim W.-I., Kang H.C., Kim S.H., Park A.H., Park E.K., Cho Y.-W., Kim S., Kim H.M., Kim J.A., Kim J., Rhee H., Kang S.-G., Kim H.D., Kim D., Kim D.S., Lee J.H. (2015). Brain somatic mutations in MTOR cause focal cortical dysplasia type II leading to intractable epilepsy. Nat. Med..

[bb0285] Liu C., Li W., Johnson G.A., Wu B. (2011). High-field (9.4T) MRI of brain dysmyelination by quantitative mapping of magnetic susceptibility. NeuroImage.

[bb0290] Lorio S., Kherif F., Ruef A., Melie Garcia L., Frackowiak R., Ashburner J., Helms G., Lutti A., Draganski B. (2016). Neurobiological origin of spurious brain morphological changes: a quantitative MRI study. Hum. Brain Mapp..

[bb0295] Lutti A., Dick F., Sereno M.I., Weiskopf N. (2014). Using high-resolution quantitative mapping of R1 as an index of cortical myelination. NeuroImage.

[bb0300] Ma D., Gulani V., Seiberlich N., Liu K., Sunshine J.L., Duerk J.L., Griswold M.A. (2013). Magnetic resonance fingerprinting. Nature.

[bb0305] Majores M., Blumcke I., Urbach H., Meroni A., Hans V., Holthausen H., Elger C.E., Schramm J., Galli C., Spreafico R., Wiestler O.D., Becker A.J. (2005). Distinct allelic variants of TSC1 and TSC2 in epilepsy-associated cortical malformations without balloon cells. J. Neuropathol. Exp. Neurol..

[bb0310] Mellerio C., Labeyrie M.-A., Chassoux F., Daumas-Duport C., Landre E., Turak B., Roux F.-X., Meder J.-F., Devaux B., Oppenheim C. (2012). Optimizing MR imaging detection of type 2 focal cortical dysplasia: best criteria for clinical practice. AJNR Am. J. Neuroradiol..

[bb0315] Mirzaa G.M., Campbell C.D., Solovieff N., Goold C.P., Jansen L.A., Menon S., Timms A.E., Conti V., Biag J.D., Olds C., Boyle E.A., Collins S., Ishak G., Poliachik S.L., Girisha K.M., Yeung K.-S., Chung B.H.Y., Rahikkala E., Gunter S.A., McDaniel S.S., Macmurdo C.F., Bernstein J.A., Martin B., Leary R.J., Mahan S., Liu S., Weaver M., Dorschner M.O., Jhangiani S., Muzny D.M., Boerwinkle E., Gibbs R.A., Lupski J.R., Shendure J., Saneto R.P., Novotny E.J., Wilson C.J., Sellers W.R., Morrissey M.P., Hevner R.F., Ojemann J.G., Guerrini R., Murphy L.O., Winckler W., Dobyns W.B. (2016). Association of MTOR mutations with developmental brain disorders, including megalencephaly, focal cortical dysplasia, and pigmentary mosaicism. JAMA Neurol..

[bb0320] Mohammadi S., Carey D., Dick F., Diedrichsen J., Sereno M.I., Reisert M., Callaghan M.F., Weiskopf N. (2015). Whole-brain in-vivo measurements of the axonal G-ratio in a group of 37 healthy volunteers. Front. Neurosci..

[bb0325] Mühlebner A., Coras R., Kobow K., Feucht M., Czech T., Stefan H., Weigel D., Buchfelder M., Holthausen H., Pieper T., Kudernatsch M., Blumcke I. (2011). Neuropathologic measurements in focal cortical dysplasias: validation of the ILAE 2011 classification system and diagnostic implications for MRI. Acta Neuropathol..

[bb0330] Nazeri A., Mulsant B.H., Rajji T.K., Levesque M.L., Pipitone J., Stefanik L., Shahab S., Roostaei T., Wheeler A.L., Chavez S., Voineskos A.N. (2016). Gray matter Neuritic microstructure deficits in schizophrenia and bipolar disorder. Biol. Psychiatry.

[bb0335] Neeb H., Ermer V., Stocker T., Shah N.J. (2008). Fast quantitative mapping of absolute water content with full brain coverage. NeuroImage.

[bb0340] Peper J.S., Brouwer R.M., Boomsma D.I., Kahn R.S., Hulshoff Pol H.E. (2007). Genetic influences on human brain structure: a review of brain imaging studies in twins. Hum. Brain Mapp..

[bb0345] Rakic P. (1988). Specification of cerebral cortical areas. Science.

[bb0350] Reeves C., Tachrount M., Thomas D., Michalak Z., Liu J., Ellis M., Diehl B., Miserocchi A., McEvoy A.W., Eriksson S., Yousry T., Thom M. (2015). Combined ex vivo 9.4T MRI and quantitative histopathological study in normal and pathological neocortical resections in focal epilepsy. Brain Pathol..

[bb0355] Rivière J.-B., Mirzaa G.M., O'Roak B.J., Beddaoui M., Alcantara D., Conway R.L., St-Onge J., Schwartzentruber J.A., Gripp K.W., Nikkel S.M., Worthylake T., Sullivan C.T., Ward T.R., Butler H.E., Kramer N.A., Albrecht B., Armour C.M., Armstrong L., Caluseriu O., Cytrynbaum C., Drolet B.A., Innes A.M., Lauzon J.L., Lin A.E., Mancini G.M.S., Meschino W.S., Reggin J.D., Saggar A.K., Lerman-Sagie T., Uyanik G., Weksberg R., Zirn B., Beaulieu C.L., Consortium F.O.R.D.G.F.C., Majewski J., Bulman D.E., O'Driscoll M., Shendure J., Graham J.M., Boycott K.M., Dobyns W.B. (2012). De novo germline and postzygotic mutations in AKT3, PIK3R2 and PIK3CA cause a spectrum of related megalencephaly syndromes. Nat. Genet..

[bb0360] Rodionov R., Bartlett P.A., He C., Vos S.B., Focke N.K., Ourselin S.G., Duncan J.S. (2015). T2 mapping outperforms normalised FLAIR in identifying hippocampal sclerosis. Neuroimage Clin..

[bb0365] Rooney W.D., Johnson G., Li X., Cohen E.R., Kim S.-G., Ugurbil K., Springer C.S. (2007). Magnetic field and tissue dependencies of human brain longitudinal 1H2O relaxation in vivo. Magn. Reson. Med..

[bb0370] Rugg-Gunn F.J., Eriksson S.H., Symms M.R., Barker G.J., Duncan J.S. (2001). Diffusion tensor imaging of cryptogenic and acquired partial epilepsies. Brain.

[bb0375] Schick V., Majores M., Engels G., Spitoni S., Koch A., Elger C.E., Simon M., Knobbe C., Blumcke I., Becker A.J. (2006). Activation of Akt independent of PTEN and CTMP tumor-suppressor gene mutations in epilepsy-associated Taylor-type focal cortical dysplasias. Acta Neuropathol..

[bb0380] Scholtens L.H., de Reus M.A., van den Heuvel M.P. (2015). Linking contemporary high resolution magnetic resonance imaging to the von Economo legacy: a study on the comparison of MRI cortical thickness and histological measurements of cortical structure. Hum. Brain Mapp..

[bb0385] Schweser F., Deistung A., Lehr B.W., Reichenbach J.R. (2011). Quantitative imaging of intrinsic magnetic tissue properties using MRI signal phase: an approach to in vivo brain iron metabolism?. NeuroImage.

[bb0390] Sim J.C., Scerri T., Fanjul Fernández M., Riseley J.R., Gillies G., Pope K., van Roozendaal H., Heng J.I., Mandelstam S.A., McGillivray G., MacGregor D., Kannan L., Maixner W., Harvey A.S., Amor D.J., Delatycki M.B., Crino P.B., Bahlo M., Lockhart P.J., Leventer R.J. (2016). Familial cortical dysplasia caused by mutation in the mammalian target of rapamycin regulator NPRL3. Ann. Neurol..

[bb0395] Song C., Schwarzkopf D.S., Kanai R., Rees G. (2015). Neural population tuning links visual cortical anatomy to human visual perception. Neuron.

[bb0400] Sotiropoulos S.N., Behrens T.E.J., Jbabdi S. (2012). Ball and rackets: inferring fiber fanning from diffusion-weighted MRI. NeuroImage.

[bb0405] Stüber C., Morawski M., Schäfer A., Labadie C., Wähnert M., Leuze C., Streicher M., Barapatre N., Reimann K., Geyer S., Spemann D., Turner R. (2014). Myelin and iron concentration in the human brain: a quantitative study of MRI contrast. NeuroImage.

[bb0410] Suemitsu L.A.Y., Yasuda C.L., Morita M.E., Beltramini G.C., Coan A.C., Bergo F., Lopes-Cendes I., Cendes F. (2014). Longitudinal analysis of hippocampal T2 relaxometry in FMTLE. Epilepsy Behav..

[bb0415] Taylor D.C., Falconer M.A., Bruton C.J., Corsellis J.A.N. (1971). Focal dysplasia of the cerebral cortex in epilepsy. J. Neurol. Neurosurg. Psychiatry.

[bb0420] Téllez-Zenteno J.F., Ronquillo L.H., Moien-Afshari F., Wiebe S. (2010). Surgical outcomes in lesional and non-lesional epilepsy: a systematic review and meta-analysis. Epilepsy Res..

[bb0425] Tofts P. (2005). Concepts: measurement and MR. Quantitative MRI of the Brain Measuring Changes Caused by Disease.

[bb0430] Vargova L., Homola A., Cicanic M., Kuncova K., Krsek P., Marusic P., Sykova E., Zamecnik J. (2011). The diffusion parameters of the extracellular space are altered in focal cortical dysplasias. Neurosci. Lett..

[bb0435] Waehnert M.D., Dinse J., Schäfer A., Geyer S., Bazin P.-L., Turner R., Tardif C.L. (2016). A subject-specific framework for in vivo myeloarchitectonic analysis using high resolution quantitative MRI. NeuroImage.

[bb0440] Wehrli F.W. (2013). Magnetic resonance of calcified tissues. J. Magn. Reson..

[bb0445] Weiskopf N., Suckling J., Williams G., Correia M.M., Inkster B., Tait R., Ooi C., Bullmore E.T., Lutti A. (2013). Quantitative multi-parameter mapping of R1, PD(*), MT, and R2(*) at 3T: a multi-center validation. Front. Neurosci..

[bb0450] Werring D.J., Clark C.A., Barker G.J., Thompson A.J., Miller D.H. (1999). Diffusion tensor imaging of lesions and normal-appearing white matter in multiple sclerosis. Neurology.

[bb0455] Whelan C.D., Alhusaini S., O'Hanlon E., Cheung M., Iyer P.M., Meaney J.F., Fagan A.J., Boyle G., Delanty N., Doherty C.P., Cavalleri G.L. (2015). White matter alterations in patients with MRI-negative temporal lobe epilepsy and their asymptomatic siblings. Epilepsia.

[bb0460] Whittall K.P., Mackay A.L., Graeb D.A., Nugent R.A., Li D.K.B., Paty D.W. (1997). In vivo measurement of T2 distributions and water contents in normal human brain. Magn. Reson. Med..

[bb0465] Widjaja E., Zarei Mahmoodabadi S., Otsubo H., Snead O.C., Holowka S., Bells S., Raybaud C. (2009). Subcortical alterations in tissue microstructure adjacent to focal cortical dysplasia: detection at diffusion-tensor MR imaging by using magnetoencephalographic dipole cluster localization 1. Radiology.

[bb0470] Winston G.P., Micallef C., Symms M.R., Alexander D.C., Duncan J.S., Zhang H. (2014). Advanced diffusion imaging sequences could aid assessing patients with focal cortical dysplasia and epilepsy. Epilepsy Res..

[bb0475] Yao B., Li T., Gelderen P., Shmueli K., Dezwart J., Duyn J. (2009). Susceptibility contrast in high field MRI of human brain as a function of tissue iron content. NeuroImage.

[bb0480] Yasin S.A., Latak K., Becherini F., Ganapathi A., Miller K., Campos O., Picker S.R., Bier N., Smith M., Thom M., Anderson G., Helen Cross J., Harkness W., Harding B., Jacques T.S. (2010). Balloon cells in human cortical dysplasia and tuberous sclerosis: isolation of a pathological progenitor-like cell. Acta Neuropathol..

[bb0485] Yasin S.A., Ali A.M., Tata M., Picker S.R., Anderson G.W., Latimer-Bowman E., Nicholson S.L., Harkness W., Cross J.H., Paine S.M.L., Jacques T.S. (2013). mTOR-dependent abnormalities in autophagy characterize human malformations of cortical development: evidence from focal cortical dysplasia and tuberous sclerosis. Acta Neuropathol..

[bb0490] Zhang H., Schneider T., Wheeler-Kingshott C.A., Alexander D.C. (2012). NODDI: practical in vivo neurite orientation dispersion and density imaging of the human brain. NeuroImage.

[bb0495] Zucca I., Milesi G., Medici V., Tassi L., Didato G., Cardinale F., Tringali G., Colombo N., Bramerio M., D'Incerti L., Freri E., Morbin M., Fugnanesi V., Figini M., Spreafico R., Garbelli R. (2016). Type II focal cortical dysplasia: ex vivo 7T magnetic resonance imaging abnormalities and histopathological comparisons. Ann. Neurol..

